# Effects of early respiratory physiotherapy on spontaneous respiratory activity of preterm infants: study protocol for a randomized controlled trial

**DOI:** 10.1186/s13063-021-05446-8

**Published:** 2021-07-26

**Authors:** Alessia Di Polito, Arianna Del Vecchio, Milena Tana, Patrizia Papacci, Anna Laura Vento, Benedetta Campagnola, Sefora Celona, Laura Cricenti, Ilaria Bastoni, Chiara Tirone, Alessandra Lio, Claudia Aurilia, Anthea Bottoni, Angela Paladini, Francesco Cota, Paola Emilia Ferrara, Gianpaolo Ronconi, Giovanni Vento

**Affiliations:** 1 Servizio Medicina Fisica e Riabilitazione, Fondazione Policlinico Universitario A. Gemelli IRCCS, Rome, Italy; 2 Dipartimento Scienze della salute della donna, del bambino e di sanità pubblica, Unità Operativa Complessa di Neonatologia, Fondazione Policlinico Universitario A. Gemelli IRCCS, Rome, Italy; 3 Dipartimento Universitario Scienze della Vita e Sanità Pubblica. Unità Operativa Complessa di Neonatologia, Fondazione Policlinico Universitario A. Gemelli IRCCS, Università Cattolica del Sacro Cuore, Largo A. Gemelli, 8, 00168 Rome, Italy

**Keywords:** Respiratory physiotherapy, Preterm infants, Spontaneous respiratory activity

## Abstract

**Background:**

Tactile maneuvers stimulating spontaneous respiratory activity in preterm infants are recommended since birth, but data on how and how often these maneuvers are applied in clinical practice are unknown. In the last years, most preterm newborns with respiratory failure are preferentially managed with non-invasive respiratory support and by stimulating spontaneous respiratory activity from the delivery room and in neonatal intensive care unit (NICU), in order to avoid the risks of intubation and prolonged mechanical ventilation.

**Methods:**

Preterm infants with gestational age < 31 weeks not intubated in the delivery room and requiring non-invasive respiratory support at birth will be eligible for the study. They will be randomized and allocated to one of two treatment groups: (1) the study group infants will be subject to the technique of respiratory facilitation within the first 24 h of life, according to the reflex stimulations, by the physiotherapist. The newborn is placed in supine decubitus and a slight digital pressure is exerted on a hemithorax. The respiratory facilitation technique will be performed for about three minutes and repeated for a total of 4/6 times in sequence, three times a day until spontaneous respiratory activity is achieved; thus, no respiratory support is required; (2) the control group infants will take part exclusively in the individualized postural care program. They will perform the technique of respiratory facilitation and autogenous drainage.

**Objective:**

To evaluate the efficacy of early respiratory physiotherapy in reducing the incidence of intubation and mechanical ventilation in the first week of life (primary outcome).

**Discussion:**

The technique of respiratory facilitation is based on reflex stimulations, applied early to preterm infant. Slight digital pressure is exerted on a “trigger point” of each hemithorax, to stimulate the respiratory activity with subsequent increase of the ipsilateral pulmonary minute ventilation and to facilitate the contralateral pulmonary expansion. This mechanism will determine the concatenation of input to all anatomical structures in relation to the area being treated, to promote spontaneous respiratory activity and reducing work of breathing, avoiding or minimizing the use of invasive respiratory support.

**Trial registration:**

UMIN-CTR Clinical Trial UMIN000036066. Registered on March 1, 2019. Protocol 1. https://www.umin.ac.jp/ctr

## Background

In the last years, most preterm newborns with respiratory failure are preferentially managed with non-invasive respiratory support and by stimulating spontaneous respiratory activity just after birth in both the delivery room and neonatal intensive care unit (NICU), in order to avoid the risks of intubation and mechanical ventilation. The latest European guidelines for the management of respiratory distress syndrome (RDS) recommend the early application of continuous positive airway pressure (CPAP) of at least 6 cm H_2_O from the delivery room in preterm infants, in order to improve and maintain adequate functional residual capacity (FRC) of the lungs [1, 2]. Some authors suggest the administration of caffeine already in the delivery room to stimulate the spontaneous respiratory activity of the preterm infants [2]. The combined use of CPAP and intravenous caffeine (and rescue administration of surfactant) is aimed to achieve and maintain adequate FRC of the preterm infant’s lung.

Tactile maneuvers stimulating spontaneous respiratory activity in preterm infants are recommended during the initial assessment since birth. Te Pas et al. observed a positive effect of the use of repetitive tactile stimulation on respiratory effort and oxygenation of preterm infants at birth [3]: rubbing the sole of the foot or the infants’ back supposedly activates proprioceptors or somatic/visceral mechanoreceptors in the thorax, respectively, which are known to stimulate spontaneous breathing [3]. These afferent somatosensory pathways are functional even before 25 weeks of gestation [4, 5]. To best of our knowledge, no randomized clinical trials have evaluated the efficacy of early and continued respiratory facilitation physiotherapy in avoiding or reducing the need for invasive respiratory support of preterm infants in NICU.

The aim of this study is to evaluate the efficacy of a respiratory facilitation physiotherapy technique according to reflex stimulations, applied within the first 24 h of life to preterm infants not intubated in the delivery room but requiring non-invasive respiratory support, and continued during hospital stay, to promote spontaneous respiratory activity, avoiding or minimizing the need for invasive respiratory support.

## Methods

The primary hypothesis of this study is a reduction in the need of mechanical ventilation (MV) in the first week of life (excluding the transient tracheal intubation performed for surfactant administration) in spontaneously breathing infants born at 24^+0^–30^+6^ weeks’ gestation receiving a respiratory facilitation maneuver according to the reflex stimulations compared to exclusive standard individualized postural care program.

The secondary outcome will be:

(i) Duration of mechanical ventilation (MV) during the hospital stay;

(ii) Duration of O_2_-therapy during the hospital stay;

(iii) Bronchopulmonary dysplasia (BPD): O_2_-dependence at 36 weeks of postmenstrual age;

(iv) Duration of non-invasive ventilation (NIV) during the hospital stay;

(v) Post-conceptional age attainment of complete respiratory autonomy (absence of any kind of respiratory support and oxygen);

(vi) Pulmonary atelectasis diagnosed on chest X-ray and/or lung ultrasound;

(vii) Length of hospital stay;

(viii) Survival.

Additional data recorded for each infant will include occurrence of: pneumothorax; pulmonary interstitial emphysema; pulmonary hemorrhage; hemodynamically significant patent ductus arteriosus requiring treatment with ibuprofen; grade 3–4 intraventricular hemorrhage [6]; periventricular leukomalacia worse than grade 2 [7]; retinopathy of prematurity [8]; necrotizing enterocolitis [9]; sepsis, defined as a positive blood culture or suggestive clinical and laboratory findings leading to treatment with antibiotics for at least 7 days despite absence of a positive blood culture; and use of systemic postnatal steroids.

This will be an unblinded mono-center randomized trial conducted at Fondazione Policlinico Universitario A. Gemelli IRCCS’s NICU. Eligible candidates will be preterm newborns with gestational age (GA) < 31 weeks not intubated in the delivery room and requiring non-invasive respiratory support. Exclusion criteria will be as follows: outborn, presence of major congenital malformations and/or genetic syndromes, fetal hydrops, inherited disorders of metabolism, persistent pulmonary hypertension of the newborn, severe circulatory shock (prolonged capillary filling, reduced strength of peripheral pulses, cool skin, lethargy, hypotension, oliguria, increasing lactate concentrations and metabolic acidosis), and other unstable clinical conditions at the time of randomization for which the newborn cannot perform the physiotherapy treatment required by the study.

All preterm infants managed in our NICU routinely follow routinely the program of individualized postural care [10–15], which include handling and positioning of the infant. Neonatal nurses and physiotherapists can play a major role in designing, modeling, and teaching positioning strategies that promote oxygen saturation stability, skeletal integrity, postural control, and sensorimotor organization. The individualized postural care program consists in alternating postures according to the needs of each individual infant. The postures are alternated in 24 h and are prone, supine, and lateral with adequate postural supports.

**Fig. 1:**
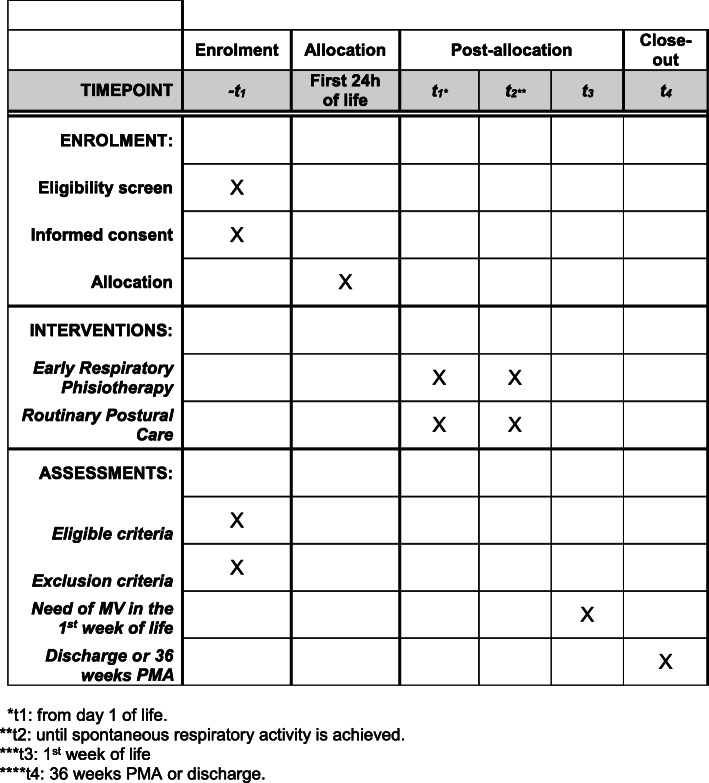
Schedule of enrolment, interventions, and assessments

Eligible infants will be allocated during the first 24 h of life to one of the two treatment groups (Fig. 1). In the study group, the physiotherapist will perform the technique of respiratory facilitation according to the reflex stimulations, experience already conducted in the neonates [16] and modified for the preterm infant. The newborn is placed in supine decubitus and a slight digital pressure is exerted on a hemithorax, more precisely between the 7th and the 8th rib (area corresponding to the insertion of the diaphragm muscle) at the level of the mammillary line, pressing from top to bottom (in the direction of the support plane) and obliquely (in the direction of the vertebral column). We will define this as a “trigger point.” Stimulating the trigger point will stimulate the respiratory activity by determining a compression on the stimulated side with consequent increase of the ipsilateral pulmonary ventilation/minute and the facilitation of the contralateral pulmonary expansion (thoracic expansion of the ribcage). The respiratory facilitation technique will be performed for about 3 min and repeated for a total of 4/6 times in sequence: 7th right hemitoraceous space for 3 min, 7th left hemithorax space for 3 min, 7th right space for a further 3 min, and 7th left space for a further 3 min. In case of secretions, the respiratory facilitation technique will be associated with the prolonged slow expired technique for the preterm infant. This program will be performed 3 times a day until complete respiratory autonomy (absence of any kind of respiratory support and oxygen) is achieved.

The control group infants will perform exclusively the individualized postural care program. They will perform the technique of respiratory facilitation and autogenous drainage modified only in the presence of clinically and radiographically established pulmonary atelectasis.

Both groups will follow the Individualized Family-Centered Developmental Care Program [17]. In particular, attention will be paid to the control of environmental stimuli such as soft lights during treatment to allow the baby to stay awake without being bothered by bright lights directly on the eyes and reduction of environmental noise (tone of the voice of the operators, noise of the monitors, cell phones).

All the interventions of the Individualized Family-Centered Developmental Care are in fact aimed at preventing and containing destabilization and neonatal discomfort considering the extreme sensitivity and vulnerability of these patients to environmental stimulation. In all newborns, the discomfort/pain measurement will be performed, using the Neonatal Pain, Agitation and Sedation Scale (NPASS) [18], according to our NICU protocol.

All patients will receive a loading dose of intravenous caffeine citrate (20 mg/kg) immediately after admission to the NICU, followed by a daily maintenance intravenous dose of 10 mg/kg. Demographic data on patient and maternal characteristics will be collected from each patient. In the two groups of infants, the following parameters will be continuously recorded and analyzed for the entire day, daily in the first week, and weekly after the first week of life: arterial oxygen saturation (SatO2) by Pulse Oximeter Masimo®; heart rate (HR); respiratory rate (RR): inspired fraction of oxygen (FiO2); episodes of apnea defined as cessation of breathing for more than 20 s, or a shorter respiratory pause associated with oxygen desaturation and/or bradycardia; episodes of bradycardia (HR< 80 bpm) or tachycardia (HR > 180 bpm); and secretions’ presence, NPASS Score, Silverman Anderson Score.

In the study group, infants will be also evaluated for vital parameters at T0, at the end of the reflex stimulations and 5 min after the end of the reflex stimulations, and evaluation of behavioral stability of children through the APIB scale (evaluation of preterm infant behavior) [19]. The APIB scale is based on behavioral articles defined according to the “Als Sinactive theory.”

The intervention in the study group will be continued also in case of intubation and start of MV during the entire hospital stay, to enhance respiratory function and favoring extubation.

Infants of both groups will receive 200 mg/kg of poractant alfa (Chiesi Farmaceutici, Parma, Italy) if they will need FiO_2_ ≥ 0.30 and CPAP of 6–7 cm H_2_O, by IN-SUR-E technique (if gestational age ≥ 28 weeks) or IN-REC-SUR-E technique [20] (if gestational age < 28 weeks), as per our NICU protocol. The indications for mechanical ventilation will be: poor oxygenation with FiO_2_ greater than 0.40 after rescue surfactant, respiratory acidosis (pCO_2_ > 65 mm Hg [8·5 kPa] and pH < 7·20), or apnea (more than four episodes of apnea per h or more than two episodes of apnea per h requiring ventilation with bag and mask), despite optimal nasal CPAP, nasal intermittent positive pressure ventilation, or bilevel positive airway pressure.

The analysis of our NICU data for the year 2017 showed how the incidence of intubation and mechanical ventilation in the first week of life in preterm infants with GA ≤ 30 weeks, not intubated in the delivery room stands at 50%. The expected effect of the intervention proposed in the trial is an absolute risk reduction of 25% (relative risk reduction of 50%) from the expected control group rate.

Considering an α error of 0.05 (two-tailed) and a study power of 80%, 66 newborns are expected to be enrolled per arm (132 total newborns); this number will be increased by 10% to take account of any dropouts.

Statistical analysis will be carried out using the “intention to treat” method; all data will be collected in a single database and analyzed to evaluate any differences between the randomized groups both for primary outcomes and for secondary outcomes. Any associations between individual dependent and independent variables will be evaluated initially and the choice of statistical methods will be decided according to the nature of the variables (categorical, continuous, rank-order) and their distribution (normal, non-normal, other). Statistical analyses will be performed using the “Stata Statistical Software: Release 15.1 software (StataCorp LP, College Station, TX).”

Infants will be randomly allocated in a 1:1 ratio to receive either technique of respiratory facilitation (intervention group) or individualized postural care (control group). Ralloc, a Stata/IC 15.1 for Windows (Stata-Corp, College Station, TX), will be used to provide a sequence of treatments randomly permuted in blocks of varying size and order. Allocation concealment will be ensured by sequentially numbered, opaque, sealed envelopes.

The study protocol was approved by the Ethics Committee of the Fondazione Policlinico Universitario A. Gemelli IRCCS, Rome, Italy, with the approval number 9108/18 ID: 1906. Written and oral information will, whenever possible, be offered to parents if their infant is likely to be eligible. Informed written consent will be signed from both the parents and sufficient time will be provided for consent.

We used the SPIRIT reporting guidelines [21].

## Safety

Safety end-point measures will include incidence, severity, and causality of reported serious adverse events (SAE), namely changes in occurrence of the expected common prematurity complications and clinical laboratory test assessments, and the development of unexpected SAEs in this high risk population. All SAEs will be followed until satisfactory resolution or until the investigator responsible for the care of the participant deems the event to be chronic or the patient to be stable. All expected and unexpected SAEs, whether or not they are attributable to the study intervention, will be reviewed by the principal investigator and by all the authors to determine if there is reasonable suspected causal relationship to the intervention. If the relationship is reasonable, SAEs will be reported to the Ethics Committee to guaranty the safety of participants.

## Discussion

The technique of respiratory facilitation is based on the reflex stimulations, applied early to the preterm infant. The slight digital pressure is exerted on a hemithorax on a so called “trigger point.” Stimulating the trigger point will stimulate the respiratory activity by determining a compression on the stimulation side with consequent increase of the ipsilateral pulmonary ventilation/minute and the facilitation of the contralateral pulmonary expansion. This mechanism will determine the concatenation of input to all anatomical structures in direct and indirect relation to the area being treated, on the basis of the mechanical-neurological-fluidic links of the human body. The above-mentioned maneuver will determine three different consequences at a distance that will positively influence the respiratory dynamics of the preterm infant. According to Sherrington’s law, in fact, a muscle subjected to a stretch will respond with a contraction of its fibers in proportion to the stretching itself. Therefore, the ipsilateral compression will cause a stretch of the muscle fibers of the diaphragm and intercostal muscles which will activate its subsequent muscular contraction and a variation of the pulmonary volume from the stimulus side with consequent increase of the pulmonary ventilation, and concurrently, there will be a greater expansion of the contralateral hematitis. From the neurological point of view, the action of the diaphragm on the phrenic nerve, on the vagus nerve, and on the orthosympathetic level should be considered. Stimulation of the VII-VIII coast will bring three different neurological information. The first at the level of D7-D8-D9 with associated neurovegetative stimulation of this area that performs its viscosymal function at the visceral level. The second concerns the vagus nerve (X n.c.) which passes through the esophagus in the esophageal hiatus and is affected by structural modifications of the diaphragm. The third in the phrenic level as a modification of the muscular mobility will determine a modification of the action of the nerve that innervates it. From a fluidic point of view, the diaphragmatic stimulation with consequent improvement of the respiratory dynamics and of the same diaphragm muscle will lead to an improvement of the caval system, since the vena cava passing through the diaphragm can better guarantee a venous return with a consequent improvement also of the portal system.

## Trial status

The trial has been registered at UMIN- CTR trial (Identifier: UMIN000036066) on March 1, 2019 protocol no. 1and it is currently recruiting study subjects. It was started on March 17, 2019. It will be completed on, approximately, May 2021.

## Data Availability

After publication of the trial report, formal request for study data should be made to the corresponding author (GV) using a data request form delineating research aims, methods, and the variables needed. Such requests will be considered by the coordinator (GV) and core team (MT, FC). If research questions and methods are considered relevant and valid, the data management department of the Policlinico Universitario A. Gemelli IRCCS will securely transfer the requested, fully anonymized data in the desired format to the party under data transfer agreement. The team will decide about co-authorships, after discussion with the interested party about this. Data requests can be submitted at any time and the data will be accessible for 12 months from publication, with possible extension considered.

## Abbreviations

NICU: Neonatal intensive care unit
MV: Mechanical ventilation
RDS: Respiratory distress syndrome
CPAP: Continuous positive airway pressure
FRC: Functional residual capacity
BPD: Bronchopulmonary dysplasia
NIV: Non-invasive ventilation
SatO_2_: Arterial oxygen saturation
HR: Heart rate
RR: Respiratory rate
FiO_2_: Inspired fraction of oxygen
NPASS: Neonatal Pain, Agitation and Sedation Scale
